# Molecular Mechanisms of Hair Follicle Development

**DOI:** 10.1155/tswj/5259055

**Published:** 2024-11-25

**Authors:** Mebrie Zemene Kinde, Tewodros Abere Mekuria, Abebe Tesfaye Gessese, Bemrew Admassu Mengistu

**Affiliations:** ^1^Department of Veterinary Biomedical Sciences, College of Veterinary Medicine and Animal Sciences, University of Gondar, Gondar, Ethiopia; ^2^Department of Veterinary Science, College of Agriculture and Natural Resource, Assosa University, Assosa, Ethiopia

**Keywords:** cytodifferentiation, hair follicle, induction, molecular mechanism, organogenesis

## Abstract

Hair is an intricate biological structure that originates from hair follicles (HFs), which are complex mini-organs embedded in the skin. Each HF undergoes continuous cycles of growth (anagen), regression (catagen), and rest (telogen), driven by intricate signaling pathways and interactions between epithelial and mesodermal cells. The development of HFs requires the interplay of several key signaling pathways, including Wnt, Shh, Notch, and BMP. The Wnt pathway is primarily involved in induction, Shh is essential for early organogenesis and later stages of cytodifferentiation, Notch signaling governs the fate of HF stem cells, and BMP plays a role in cytodifferentiation. Hair health is closely associated with psychological well-being and personal distress. While hair loss (alopecia) does not impact biological health, it significantly affects social well-being. Therefore, a deep understanding of the molecular mechanisms underlying HF development is crucial for developing treatments for hair-related problems and improving hair health. This knowledge has led to significant advancements in therapeutic applications, particularly in treating hair loss disorders, enhancing wound healing, and developing cosmetic treatments. This paper aims to review the molecular mechanisms involved in HF development, with an emphasis on their potential impact on human health and well-being.

## 1. Introduction

Hair is an intricate biological structure composed of dead epithelial cells called keratinocytes, which condense into fibers with remarkable tensile strength [1, 2]. As a distinguishing characteristic of mammals, hair plays several vital roles, including regulating body temperature, providing a barrier against physical injury and infectious agents, dispersing sweat and sebum, enhancing sensory perception, facilitating social interactions, and offering camouflage [3, 4]. In human societies, hair holds immense psychosocial importance, influencing personal identity, cultural norms, and social interactions. Conversely, hair loss or excessive hair growth due to various medical conditions can have profound psychological and emotional effects, underscoring the clinical significance of hair biology [4].

Hair shafts (HSs) originate from hair follicles (HFs), which are complex mini-organs embedded in the skin. The HF structure includes the pilosebaceous unit, sebaceous and apocrine glands, and the arrector pili muscle. A mature HF is a sophisticated structure organized into several concentric layers of epithelial cells, known as root sheaths, which surround the HS [5]. HFs contain multipotent stem cells (MSCs) capable of regenerating all skin derivatives, giving them the remarkable ability to self-renew throughout an individual's life, continually producing new HSs. Each HF undergoes continuous cycles of growth (anagen), regression (catagen), and rest (telogen), driven by intricate signaling pathways and interactions between epithelial and mesenchymal cells (MCs) [4, 6]. During morphogenesis, a critical phase in HF development, ectodermal HF stem cells give rise to all epithelial components of the HF, including sebaceous and apocrine glands, while mesodermal cells form the follicular dermal papilla and connective tissue sheath [4, 7, 8].

HF development begins during fetal and perinatal skin formation, and intriguingly, de novo HF formation can also occur in adults after skin injury. Both embryonic HF development and the postnatal hair cycle are conserved processes regulated by a series of interactive signals between epithelial and MCs [9]. Several signaling pathways, including the wingless (Wnt), hedgehog, transforming growth factor-*β* (TGF-*β*), fibroblast growth factor (FGF), and tumor necrosis factor (TNF) families, are involved in HF development and its regular cycles. HF morphogenesis encompasses three stages: induction, organogenesis, and cytodifferentiation. During induction, Wnt-mediated signaling from MCs leads to the formation of epidermal thickenings called placodes. In the organogenesis stage, interactions between placode epithelial cells and underlying dermal fibroblasts form a dermal condensate and drive epithelial cells to grow downward into the dermis. In the cytodifferentiation stage, the dermal condensate becomes surrounded by follicular epithelial cells, forming the dermal papilla, which uses growth factors and morphogens to guide the ectodermal shaping of the HF [10]. Various signaling mechanisms identified in mouse models, including spontaneous mutants and genetically engineered mice, have illuminated early HF morphogenesis [11].

Hair health is closely associated with personal distress and psychological well-being. While hair loss (alopecia) does not impact biological health, it significantly affects social well-being. Therefore, a deep understanding of the molecular mechanisms underlying HF development is crucial for developing treatments for hair-related problems and improving hair health. Advances in understanding the hair growth cycle and the role of dihydrotestosterone (DHT) in HF miniaturization have led to the development of drugs like minoxidil, which prolongs the anagen phase, and finasteride, which inhibits DHT conversion, thereby reducing hair loss and promoting regrowth [12, 13]. In treating alopecia areata, an autoimmune disorder, Janus kinase (JAK) inhibitors such as tofacitinib and ruxolitinib, block inflammatory pathways, leading to significant hair regrowth [14, 15]. Furthermore, understanding signaling pathways such as Wnt/*β*-catenin and BMP has enabled the bioengineering of HFs for potential baldness treatment, with techniques such as 3D bioprinting and scaffold-based approaches being explored [16, 17].

Insights into HF development have also enhanced stem cell therapies, regenerative medicine, wound healing, and cosmetic treatments. Studies on HF stem cell niches and signals, such as TGF-*β* and FGF, have led to therapies like microneedling combined with growth factors, and autologous cell-based therapy shows promise in clinical trials [18]. Incorporating HF-derived cells in skin grafts improves vascularization and healing of chronic wounds and burns [19]. Advances in HF biology have also led to the development of topical treatments and dietary supplements that support hair growth and health [20, 21]. Personalized therapies based on genetic and environmental factors influencing hair growth aim to tailor treatments for more effective outcomes and reduced side effects [20]. This paper aims to review the molecular mechanisms involved in HF development, emphasizing their potential impact on human health and well-being.

## 2. Development of HF

**Figure 1:**
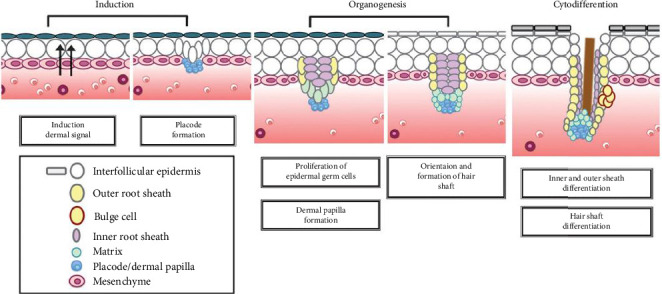
Embryonic developmental stages of the hair follicle [22].

**Figure 2:**
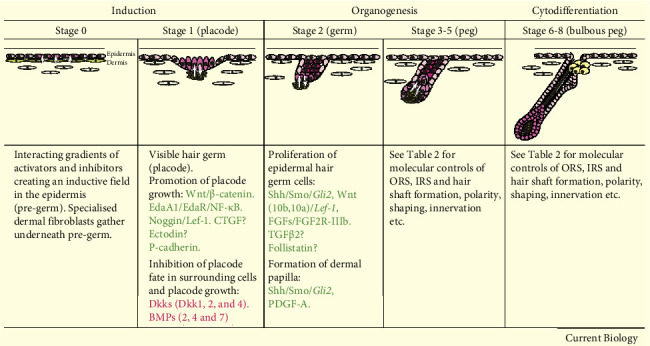
Stages of HF development with molecules and signaling pathways [25].

The development of HF during embryogenesis involves three stages (Figure 1) including induction, organogenesis, and cytodifferentiation, which are regulated through several signaling pathways [23, 24] (Figure 2).

### 2.1. HF Induction

Localized thickening of epidermis which is also known as hair placode is formed in HF induction. The placode is developed from a stem cell having G protein coupled receptor 6 [26] (Figure 2). The very first signal necessary for induction is Wnt [8]. Wnt is categorized as primary which includes Wnts 3, 4, and 6 and is responsible for HF initiation and secondary such as Wnts 2, 7b, 10a, and 10b which are involved in the development of HF. Ligands of epidermal Wnt stabilize and regulate dermal *β*-catenin signaling, which results in HF initiation. Transgenic mice which express stabilized *β*-catenin in their epidermis form excessive HFs with premature and expanded placode development. In contrary, the ablation of *β*-catenin in the epidermis results in a failure of HF initiation. The activation of *β*-catenin expression is sequential, which is started in upper dermis continued with the hair placode epithelium and end within the dermal condensates. Dermal signals regulate placode, which, in turn, regulates assembling of underlying fibroblast [27–29].

The signaling pathways of epithelial Wnt/*β*-catenin and EDA/EDAR/NF-*κ*B signaling pathways play very crucial role in the initiation of HF and maintenance of primary placodes [23]. Wnt/*β*-catenin controls the expression of EDA as well as its receptor, EDAR. EDAR signaling is found to be essential in refining Wnt/*β*-catenin pattern during primary placodes induction. It is also revealed to suppress signals of BMP, which inhibits the formation of placode [30].

Irregular placode borders with fused or string shaped patches of cells were found in a condition where NF-*κ*B or Edar signaling is absent. This shows that NF-*κ*B and Edar play a significant role in determining the arrangement of hair placode borders. NF-*κ*B refines the hair placode borders indirectly by controlling Wnt signaling. Wnt signaling inhibitor, Dkk4, expression is controlled by NF-*κ*B besides to LEF/TCF/*β*-catenin. Competition between stimulating signals of placode formation such as Wnt10b and *β*-catenin and inhibitory signals such as Dkk4 determines the establishment of regular hair placode array [31].

Condensation of dermal fibroblasts is occurred after placode formation. FGF20 is found to mediate the condensation which is expressed in hair placodes. The epithelial Eda/Edar and Wnt/*β*-catenin signaling are necessary to induce the expression of FGF20 and as to facilitate the condensation of the underlying dermis. During HF development, FGF20 regulates primary as well as secondary condensation. FGF signals show a role during multiple stages of HF development. The deletion of FGFR2B shows slow HF development [32, 33]. The induction of HF has also been inhibited via FGF7 [34].

Further HF initiation at the placode stage prerequisites a downregulation of keratinocyte growth factor (KGF) and epidermal growth factor (EGF) molecules, which is achieved only through downregulating their receptors (EGFR and FGFR IIIb). Therefore, the ligands will remain unpaired during the course of the initiation [33].

### 2.2. HF Organogenesis

The creation of the hair germ, also known as the follicular bud, begins with the condensation of MCs beneath the placode during organogenesis (Figure 2). The hair germ then multiplies and invades the dermis, forming the hair peg and the bulbous peg. Placode proliferation and dermal condensation happen at the same time, and significant keratinocyte proliferation signals the start of organogenesis. The proliferation of the mesenchymal condensate and the growth of epithelial cells downward into the dermis are organized by a complex interaction of signals conveyed between the epithelial placode and the underlying mesenchymal condensate [23, 28].

In order for HF induction to proceed to organogenesis stage, BMP should have to be blocked. Dermal Noggin was found to inhibit BMP and regulate HF epithelial induction through Lef1 [35, 36].

Shh signaling pathway has an important role in mediating early hair organogenesis which is revealed in Shh knockout mice [37]. Placode cells proliferate and produce downgrowths following induction, thanks to two central signaling pathways, namely, Shh and Pdgf. As development progresses, Shh is first expressed in the developing placode and later confined to the tip of the downgrowing bulb in contact with the DP [9].

BMP-mediated inhibition requires the production of dermal Noggin. BMP inhibition mediated by dermal Noggin was shown to be required for persistent Shh expression [38]. Shh Noggin activation is a complicated process. Cross-interaction between epithelial and MCs is required for both Noggin expression and signal exchange. Epithelial Shh expression, epithelium laminin-511, epithelial platelet-derived growth factor (PDGF), dermal integrin, and dermal expression of PDGFR are all required for noggin expression. Laminin-511, generated from epithelial cells, interacts with mesenchymal integrin to promote the development of primary cilia. The primary cilia, in turn, mediates epithelial-derived Shh to activate downstream Shh effectors such patched, smoothened, and Gli to commence signaling. Epithelial PDGF activates mesenchymal PDGFR, which in turn activates dermal cells to secrete Noggin when paired with Shh signaling. Noggin suppresses BMP signaling in epithelial cells, allowing for the production of Lef1 and thus the release of inhibited epithelial Wnt signaling. As a result, laminin-511 enhances both mesenchymal Shh signaling and epithelial Shh expression via Noggin-mediated BMP inhibition [37].

In the lack of signals and Snail expression, HF development will be hampered. TGF-2 signaling is required for Snail induction as well as the Ras-mitogen-activated protein kinase (MAPK) pathway activation in the bud. Snail is a protein that controls cell growth and cell adhesion. As a result, the TGF-2 signaling pathway accurately controls epithelial proliferation, junctional remodeling, and bud formation, allowing hair morphogenesis to proceed normally [39].

### 2.3. Cytodifferentiation

The edges of the down-growing follicular epithelium progressively surround the dermal condensate to form the DP. Signals from the DP will induce the adjacent epithelial cells to differentiate into the inner root sheath (IRS), a structure in which the future HS will develop (Figure 2). After the IRS is formed, the epithelial cells surrounding the DP (also known as matrix cells) commence differentiating into distinct lineages to form the different components of the HS that will grow inside the IRS and in the end protruding through the epidermis. Cytodifferentiation is characterized by the development of different parts of HF. Several signaling molecules have been identified to regulate this process. The differentiation of IRS is regulated by the transcription factors (TFs) named Gata3 and Cutl while HS differentiation is regulated by BMP signaling and TFs such as Msx2, FoxN1, and Hoxc13 [40–44].

In dermal papillae, Notch1 binds to the RBP-Jk binding site on the promoter region. This binding activates the expression of Wnt5a that mediates Notch signaling by means of assisting FoxN1 gene expression. FoxN1 is required for HF keratinocyte development as well as for signaling the particular transfer of pigment from melanocytes to hair cortical keratinocytes. The underlying mesenchyme regulates HF differentiation via the Notch-CSl pathway, which includes Wnt5a and FoxN1 mediators [45, 46].

DP through the expression of Sox2 controls differentiation of HS precursor. Sox2 directly targets BMP6 and Sostdc1 so as to lead to parallel upregulation and downregulation of BMP6 and Sostdc1, respectively. The role of BMP6 is to inhibit cell migration. Sostdc1 is found to be a potent BMP inhibitor. Therefore, Sox2 regulates the growth of hair by directing the migration of precursor cells through BMP-mediated interaction of epithelium and mesenchymal crosstalk [47].

Another important signaling pathway identified in HF differentiation is BMP/BMPRIA. BMPRIA is the only known BMP receptor expressed in HFs. BMP has a great role in epithelial stem cell maintenance and progenitor differentiation. The effect of BMP is mediated via its receptor, BMPRIA, which is critical for precursor cell differentiation in the IRS and HS. GATA3 is induced by BMP4, and it acts as a terminal differentiation factor. BMP levels in turn are maintained by GATA3 establishing a feedback loop. Therefore, the activation of BMPRIA stimulates the differentiation of IRS progenitor cells through GATA3. HS differentiation is regulated by Wnt signaling via BMPRIA signaling. Successive inhibition and activation of BMPRIA in precursor cells maintain enough Lef1 and stabilized *β*-catenin to activate the HF-specific keratin and generate HS [48]. It was shown that firstly active epithelial BMP4 activates Msx1 and a number of other TFs, which in turn induce the expression of dermal or mesenchymal BMP4 [48].

## 3. Stem Cells and Their Role in HF Development

HF stem cells (HFSCs) in the bulge are a multipotent adult stem cell population. They can periodically give rise to new HFs and even regenerate the epidermis and sebaceous glands during wound healing [49, 50]. Epithelial, melanocyte, mesenchymal, and neural stem cells are abundant in the HF and its connective tissue sheath. The bulging part of the HF contains adult epithelial HF stem cells [6]. The activation of HF stem cells occurs during the start of anagen, and the stem cell begins to divide. The daughter cells that have been divided travel to the follicle's base, where they multiply and become transiently active matrix cells. The differentiation potential of stem cells in the bulge to all cell lineages of the adult HF was validated in vivo labeling and transplantation investigations [51].

The occurrence of severe inflammatory damage to the bulge results in cicatricial alopecia, which is permanent. Bulge stem cells only contribute to HF maintenance and repairment if the skin is not damaged. They can, however, also produce sebaceous glands and the interfollicular epidermis [52].

Despite the fact that HF stem cells have varied functions, including the potential to generate HFs, sebaceous glands, and interfollicular epidermis, they are susceptible to multiple genetic mutations due to their long lifespan, and their quiescent nature may facilitate carcinogen retention, making them more prone to tumor development, for instance, carcinoma of basal cell [53].

Wnt pathway activation genes are discovered to be repressed in bulge stem cells, while Wnt inhibitors such as Srfp1, Dab2, and TCF3 are shown to be overexpressed when compared to nonbulge keratinocytes. This is in line with the theory that Wnt signaling causes epithelial HF stem cells to develop and take on a hair-like appearance. BMP signaling is required for HF stem cell dormancy/quiescence [54]. It was discovered that mice lacking BMPR1a had a constant activation and aberrant proliferation of HF stem cells, which leads to the loss of slow-cycling cells. As expected, these mice reveal increased and abnormal levels of Lef-1 and stabilized *β*-catenin in the stem cell niche, indicating that BMP signaling may be important for slowing the cell cycle and maintaining the HF stem cell population by preventing the activation of the Wnt pathway [55].

Slow cycling ensures stem cell survival, which is regulated by low levels of c-myc and the expression of the TFs LHX2 and Sox9. Inhibition of BMP by antagonists such as Wnts and stabilized *β*-catenin [54] as well as elevated levels of c-myc and Runx1 4 is required for stem cell activation during the telogen–anagen transition.

Before birth, epithelial HF stem cells as well as their progeny exist at the earliest stages of HF development. Sox9 expression in stem cells was discovered to be essential for the creation of a proper matrix, outer root sheath, bulge, and sebaceous gland after birth. On contrary, Lhx2 expression in stem cells is present in the early placode and germ, but it disappears later in HF development. These cells are described as temporary amplifying cells which are involved in prenatal HF initiation and development [56].

## 4. Molecular Regulation of HF Characteristics

### 4.1. Polarity of HF

The growth of HF in the skin occurs at an angle pointing from front to back. This polarity could be influenced by Shh, which has an asymmetric expression pattern in hair and feather follicles [51]. The overexpression of Shh in embryonic chick skin also induces the production of larger feather buds that have lost their natural orientation, according to another study [52].

It was observed that the overexpression of Wnt7a or stabilized b-catenin in embryonic chick skin and overexpression of Lef1 or stabilized b-catenin in transgenic mouse skin result in altered follicular polarity and be able to induce symmetrical expression of Shh in the follicle, suggesting that WNT signals may lie upstream of Shh in controlling polarity [53].

### 4.2. Control of HF Shape

It was observed that a mutation in the genes encoding TGF-*α*, the TGF receptor and the TF ETS2, cause different HF architecture and wavy hair, demonstrating that these factors play significant roles in regulating the shape of HFs [54]. Therefore, anything that can affect those genes expression may contribute for the numerous variations in hair texture seen among human populations.

### 4.3. Mechanical Integrity of HSs

HS rigidity is an important aspect of hair growth cycle in which a little deviation can influences hair shape. Many genetic changes are related to hair fragility but their molecular mechanism in this regard is not understood well. Desmosomes that anchor cell to basement membrane is very critical for the integrity of epithelial tissues. Hence, a mutation of a transmembrane protein cadherin (which connects the cell to the basement membrane in desmosomes), in developing hair may show a decrease in HS rigidity. The bald mice with a characteristic of broken and twisted HSs found to suffer from non-functional desmoglein 3 [55]. Similarly, increased HS fragility was observed in a mutation of desmoglein four in lanceolate mice [56].

Proper keratinization of the shaft appears to be the key to form a flexible but almost unbreakable hair. Hence, the presence of sufficient and balanced keratin synthesis is mandatory. Even though hair brittleness may occur due to various reasons, the analysis of mice with a fragile hair phenotype shows the importance of genetics in controlling keratinization. Terminal differentiation process in HF was shown to be disturbed by Hoxc13 and Hoxc13 deficient mice showed significant hair fragility [56]. It was also observed that over-expression of Wnt3 in the outer root sheath was able to induce the fractures of HSs [57].

## 5. Application of HF Development in Therapeutics

Hair health is associated with personal distress and psychological well-being. Even though hair loss (alopecia) does not affect humans' biological health, it affects an individual's social well-being. So, treatment for hair problems and improving hair health are obligatory [58]. Understanding HF development has led to significant advancements in therapeutic applications, particularly in treating hair loss disorders, enhancing wound healing, and developing cosmetic treatments. One notable example is in the treatment of androgenetic alopecia (pattern baldness). Research into the hair growth cycle and the role of DHT in HF miniaturization has led to the development of drugs such as minoxidil, which prolongs the anagen phase of hair growth, and finasteride, which inhibits the conversion of testosterone to DHT, thereby reducing hair loss and promoting regrowth [12, 13].

For alopecia areata, an autoimmune disorder causing hair loss, understanding the immune mechanisms involved in HF cycling has been crucial. This knowledge has led to the use of JAK inhibitors such as tofacitinib and ruxolitinib, which block the inflammatory pathways that cause HF destruction, resulting in significant hair regrowth in affected individuals [14, 15].

In regenerative medicine, understanding the signaling pathways and cellular interactions necessary for HF formation has enabled scientists to bioengineer HFs. For example, research on the Wnt/*β*-catenin and BMP signaling pathways has been used to create new HFs in vitro by combining epithelial and MCs in specific culture conditions. These bioengineered follicles can potentially be transplanted to treat baldness. Techniques such as 3D bioprinting and scaffold-based approaches are being explored to construct complex tissue structures that support hair growth [16, 17, 59]. Several plant-derived chemicals such as Loliolide, Malva verticilata and Thuja orientalis have also been reported to promote hair growth by activating Wnt/*β*-catenin signaling in various in vitro and in vivo studies [60–62].

Stem cell therapies have also benefited from insights into HF development. Studies on the niches and signals that regulate HF stem cells, such as TGF-*β* and FGF, have led to treatments that activate these cells. Microneedling combined with growth factors is one such therapy that stimulates dormant stem cells, promoting hair regrowth. Mesenchymal stem cell-derived signaling and growth factors obtained by platelets influence hair growth through cellular proliferation to prolong the anagen phase (FGF-7), induce cell growth (ERK activation), stimulate HF development (*β*-catenin), and suppress apoptotic cues (Bcl-2 release and Akt activation) [63]. Additionally, autologous cell-based therapy, which involves the extraction, expansion, and reinjection of a patient's own HF stem cells, has shown promise in clinical trials for treating hair loss disorders [18, 59, 64].

Understanding HF development has also improved wound healing and skin grafting techniques. HFs play a crucial role in skin homeostasis and wound healing, and incorporating HF-derived cells in skin grafts has been shown to enhance vascularization and healing of chronic wounds and burns [19, 58, 65]. There is an increasing number of clinical and experimental studies exploring the mechanisms by which HFs contribute to skin recovery. Mouse tracing experiments demonstrated that after skin injury, epithelial stem cells migrate out of the follicle to support wound re-epithelialization, while the MCs from the HF are mobilized to migrate into the wound bed and contribute to the repair of the dermis. Accordingly, there is a significant delay in wound healing in the absence of HFs, observed both in experimental studies in mice and in humans during clinical practice [66].

In the realm of cosmetic and anti-aging treatments, detailed knowledge of HF biology has led to the development of advanced topical treatments containing growth factors, peptides, and other bioactive molecules that support hair growth and health. Moreover, dietary supplements enriched with vitamins and minerals essential for HF function, such as biotin and zinc, are widely used to maintain hair health [20, 21, 67–71].

Furthermore, insights into HF biology are guiding the development of personalized therapies. By understanding the genetic and environmental factors influencing hair growth, researchers aim to tailor treatments to individual patients. This personalized approach may lead to more effective outcomes and reduced side effects compared to traditional one-size-fits-all approaches [20, 65]. For instance, advances in tissue engineering and stem cell research are paving the way for innovative therapies that can regenerate HFs and stimulate hair growth [65]. Additionally, understanding the dynamics of stem cell niches within HFs is crucial for developing these personalized treatments [72].

## 6. Potential Challenges

Applying the knowledge of HF development to therapeutic interventions faces several challenges and limitations that must be addressed for successful clinical outcomes. One significant challenge lies in the complexity of HF biology itself. HF development is regulated by intricate molecular pathways and interactions between epithelial cell and MC, which can vary depending on the anatomical location and individual genetics [16]. This complexity makes it challenging to replicate the natural processes of hair growth and regeneration artificially.

Another challenge is the heterogeneity of hair loss disorders among patients. Androgenetic alopecia, alopecia areata, and other forms of hair loss can have different underlying causes and mechanisms, necessitating personalized treatment approaches [13, 15]. What works for one type of hair loss may not be effective for another, requiring a tailored approach based on individual genetic predispositions and immune profiles.

Furthermore, the clinical translation of bioengineered HFs and stem cell therapies faces regulatory and safety hurdles. While promising in preclinical studies, bioengineered follicles must demonstrate long-term functionality, including appropriate integration into the host tissue and sustained hair growth, to be considered viable treatments [17, 18]. Safety concerns also surround stem cell therapies, particularly regarding potential tumorigenicity and the need for rigorous quality control and monitoring of injected cells.

Additionally, the financial cost of advanced hair loss treatments remains a significant barrier to widespread adoption. Many innovative therapies, such as JAK inhibitors for alopecia areata or personalized stem cell treatments, can be prohibitively expensive for patients without adequate insurance coverage or healthcare support [14, 20].

Lastly, ethical considerations arise in the development and application of HF therapies, particularly in the context of genetic modification and manipulation of human cells. Issues such as informed consent, equitable access to treatments, and long-term implications for patients' health and well-being must be carefully evaluated [19].

Addressing these challenges requires multidisciplinary collaboration between scientists, clinicians, regulatory authorities, and patient advocacy groups to ensure that advancements in understanding HF development translate into safe, effective, and accessible therapeutic interventions.

## 7. Conclusion

Development of HF requires a complex series of reciprocal signals between the dermal epithelium and the underlying dermal. The precise initiating stimulus though has not been to be identified, the phases can be morphologically classified as induction, organogenesis and cytodifferentiation. Having enough understanding of molecules within the body and signaling pathways that are responsible for HF formation and also for hair growth is very crucial to achieve therapeutic goals in the case of hair loss. The therapeutic aims include the ability to create new HFs, being able to change the characteristics (such as size or shape) of existing follicles, and to alter hair growth in existing follicles. This can be succeeded through specifically targeting the key molecules involved in HF development regulation, perhaps even to the point of de novo induction of HF in adult human skin. Currently, some promising medications have emerged from the insights gained from the signaling pathways involved in HF, providing some success in the treatment of HF abnormalities. Moreover, the HF and the mesenchyme surrounding it are nowadays accepted as potential sources of MSC populations, which raises the hope that stem cells in adult human HFs might become applicable in regenerative medicine. Striking advances have been made recently in our understanding of HF development; however, its application in stem cell based therapy for treatment of hair abnormalities remained limited because of carcinogenesis risk. Therefore, in order to implement our knowledge on the molecular mechanisms of HF development, further, more in-depth research is necessary.

## Data Availability

The data supporting this review are from previously reported studies, which have been cited.

## References

[1] Randall V. A., Botchkareva N. V. (2009). Chapter 1—The Biology of Hair Growth. *Personal Care and Cosmetic Technology, Cosmetics Applications of Laser and Light-Based Systems*.

[2] Hoover E., Alhajj M., Flores J. L., Physiology H. (2024). *StatPearls*.

[3] Dawson J., Murina A. T., Li B. S., Maibach H. I. (2021). Biology of Hair. *Ethnic Skin and Hair and Other Cultural Considerations. Updates in Clinical Dermatology*.

[4] Pelissier-Alicot A. L., Kintz P., Salomone A., Vincenti M. (2023). *Perspectives and Challenges of Hair Analysis*.

[5] Park S. (2022). Hair Follicle Morphogenesis During Embryogenesis, Neogenesis, and Organogenesis. *Frontiers in Cell and Developmental Biology*.

[6] Houschyar K. S., Borrelli M., Tapking C. (2020). Molecular Mechanisms of Hair Growth and Regeneration: Current Understanding and Novel Paradigms. *Dermatology*.

[7] Fuchs E. (2007). Scratching the Surface of Skin Development. *Nature*.

[8] Paus R., Cotsarelis G. (1999). The Biology of Hair Follicles. *New England Journal of Medicine*.

[9] Millar S. E. (2002). Molecular Mechanisms Regulating Hair Follicle Development. *Journal of Investigative Dermatology*.

[10] Schmidt-Ullrich R., Paus R. (2005). Molecular Principles of Hair Follicle Induction and Morphogenesis. *BioEssays*.

[11] Mou C., Jackson B., Schneider P., Overbeek P. A., Headon D. J. (2006). Generation of the Primary Hair Follicle Pattern. *Proceedings of the National Academy of Sciences of the United States of America*.

[12] Kaiser M., Abdin R., Gaumond S. I., Issa N. T., Jimenez J. J. (2023). Treatment of Androgenetic Alopecia: Current Guidance and Unmet Needs. *Clinical, Cosmetic and Investigational Dermatology*.

[13] Gupta A. K., Charrette A. (2014). The Efficacy and Safety of 5*α*-Reductase Inhibitors in Androgenetic Alopecia: A Network Meta-Analysis and Benefit-Risk Assessment of Finasteride and Dutasteride. *Journal of Dermatological Treatment*.

[14] Kennedy Crispin M., Ko J. M., Craiglow B. G. (2016). Safety and Efficacy of the JAK Inhibitor Tofacitinib Citrate in Patients With Alopecia Areata. *JCI Insight*.

[15] Mackay-Wiggan J., Jabbari A., Nguyen N (2016). Oral Ruxolitinib Induces Hair Regrowth in Patients With Moderate-To-Severe Alopecia Areata. *JCI Insight*.

[16] Yang C. C., Cotsarelis G. (2010). Review of Hair Follicle Dermal Cells. *Journal of Dermatological Science*.

[17] Toyoshima K. E., Asakawa K., Ishibashi N. (2012). Fully Functional Hair Follicle Regeneration Through the Rearrangement of Stem Cells and Their Niches. *Nature Communications*.

[18] Fukuoka H., Suga H. (2015). Hair Regeneration Treatment Using Adipose-Derived Stem Cell-Conditioned Medium: Follow-Up With Trichograms. *Eplasty*.

[19] Diegelmann R. F., Evans M. C. (2004). Wound Healing: An Overview of Acute, Fibrotic and Delayed Healing. *Frontiers in Bioscience*.

[20] Trüeb R. M. (2016). Serum Biotin Levels in Women Complaining of Hair Loss. *International Journal of Trichology*.

[21] Almohanna H. M., Ahmed A. A., Tsatalis J. P., Tosti A. (2019). The Role of Vitamins and Minerals in Hair Loss: A Review. *Dermatologic Therapy*.

[22] Forni M. F., Trombetta-Lima M., Sogayar M. C. (2012). Stem Cells in Embryonic Skin Development. *Biological Research*.

[23] Daszczuk P., Mazurek P., Pieczonka T. D., Olczak A., Boryń Ł M., Kobielak K. (2020). An Intrinsic Oscillation of Gene Networks Inside Hair Follicle Stem Cells: An Additional Layer that Can Modulate Hair Stem Cell Activities. *Frontiers in Cell and Developmental Biology*.

[24] Rishikaysh P., Dev K., Diaz D., Qureshi W. M. S., Filip S., Mokry J. (2014). Signaling Involved in Hair Follicle Morphogenesis and Development. *International Journal of Molecular Sciences*.

[25] Schneider M. R., Schmidt-Ullrich R., Paus R. (2009). The Hair Follicle as a Dynamic Miniorgan. *Current Biology*.

[26] Biggs L. C., Mikkola M. L. (2014). Early Inductive Events in Ectodermal Appendage Morphogenesis. *Seminars in Cell & Developmental Biology*.

[27] Zhang Y., Andl T., Yang S. H (2008). Activation of *β*-Catenin Signaling Programs Embryonic Epidermis to Hair Follicle Fate. *Development*.

[28] Huelsken J., Vogel R., Erdmann B., Cotsarelis G., Birchmeier W. (2001). *β*-Catenin Controls Hair Follicle Morphogenesis and Stem Cell Differentiation in the Skin. *Cell*.

[29] Hardy M. H. (1992). The Secret Life of the Hair Follicle. *Trends in Genetics*.

[30] Pummila M., Fliniaux I., Jaatinen R (2007). Ectodysplasin Has a Dual Role in Ectodermal Organogenesis: Inhibition of Bmp Activity and Induction of Shh Expression. *Development*.

[31] Sick S., Reinker S., Timmer J., Schlake T. (2006). WNT and DKK Determine Hair Follicle Spacing Through a Reaction-Diffusion Mechanism. *Science*.

[32] Petiot A., Conti F. J., Grose R., Revest J. M., Hodivala-Dilke K. M., Dickson C. A. (2003). A Crucial Role for Fgfr2-IIIb Signalling in Epidermal Development and Hair Follicle Patterning. *Development*.

[33] Richardson G. D., Bazzi H., Fantauzzo K. A (2009). KGF and EGF Signalling Block Hair Follicle Induction and Promote Interfollicular Epidermal Fate in Developing Mouse Skin. *Development*.

[34] Ohuchi H., Tao H., Ohata K (2003). Fibroblast Growth Factor 10 Is Required for Proper Development of the Mouse Whiskers. *Biochemical and Biophysical Research Communications*.

[35] Botchkarev V. A., Botchkareva N. V., Roth W (1999). Noggin Is a Mesenchymally Derived Stimulator of Hair-Follicle Induction. *Nature Cell Biology*.

[36] Jamora C., DasGupta R., Kocieniewski P., Fuchs E. (2003). Links Between Signal Transduction, Transcription and Adhesion in Epithelial Bud Development. *Nature*.

[37] Gao J., DeRouen M. C., Chen C. H (2008). Laminin-511 Is an Epithelial Message Promoting Dermal Papilla Development and Function During Early Hair Morphogenesis. *Genes and Development*.

[38] Blanpain C., Fuchs E. (2006). Epidermal Stem Cells of the Skin. *Annual Review of Cell and Developmental Biology*.

[39] Jamora C., Lee P., Kocieniewski P (2004). A Signaling Pathway Involving TGF-Β2 and Snail in Hair Follicle Morphogenesis. *PLoS Biology*.

[40] Godwin A. R., Capecchi M. R. (1998). Hoxc13 Mutant Mice Lack External Hair. *Genes and Development*.

[41] Meier N., Dear T. N., Boehm T. (1999). Whn and mHa3 Are Components of the Genetic Hierarchy Controlling Hair Follicle Differentiation. *Mechanisms of Development*.

[42] Kulessa H., Turk G., Hogan B. L. (2000). Inhibition of Bmp Signaling Affects Growth and Differentiation in the Anagen Hair Follicle. *The EMBO Journal*.

[43] Tkatchenko A. V., Visconti R. P., Shang L (2001). Overexpression of Hoxc13 in Differentiating Keratinocytes Results in Downregulation of a Novel Hair Keratin Gene Cluster and Alopecia. *Development*.

[44] Mecklenburg L., Nakamura M., Sundberg J. P., Paus R. (2001). The Nude Mouse Skin Phenotype: The Role of Foxn1 in Hair Follicle Development and Cycling. *Experimental and Molecular Pathology*.

[45] Weiner L., Han R., Scicchitano B. M. (2007). Dedicated Epithelial Recipient Cells Determine Pigmentation Patterns. *Cell*.

[46] Bray S. J. (2006). Notch Signalling: A Simple Pathway Becomes Complex. *Nature Reviews Molecular Cell Biology*.

[47] Rendl M., Polak L., Fuchs E. (2008). BMP Signaling in Dermal Papilla Cells Is Required for Their Hair Follicle-Inductive Properties. *Genes and Development*.

[48] Taylor G., Lehrer M. S., Jensen P. J., Sun T. T., Lavker R. M. (2000). Involvement of Follicular Stem Cells in Forming Not Only the Follicle but Also the Epidermis. *Cell*.

[49] Lee J. H., Choi S. (2024). Deciphering the Molecular Mechanisms of Stem Cell Dynamics in Hair Follicle Regeneration. *Experimental and Molecular Medicine*.

[50] Xing Y. Z., Guo H. Y., Xiang F., Li Y. H. (2024). Recent Progress in Hair Follicle Stem Cell Markers and Their Regulatory Roles. *World Journal of Stem Cells*.

[51] Blanpain C., Lowry W. E., Geoghegan A., Polak L., Fuchs E. (2004). Self-Renewal, Multipotency, and the Existence of Two Cell Populations Within an Epithelial Stem Cell Niche. *Cell*.

[52] Ting-Berreth S. A., Chuong C. (1996). Sonic Hedgehog in Feather Morphogenesis: Induction of Mesenchymal Condensation and Association With Cell Death. *Developmental Dynamics*.

[53] Gat U., DasGupta R., Degenstein L., Fuchs E. (1998). De Novo Hair Follicle Morphogenesis and Hair Tumors in Mice Expressing a Truncated *β*-Catenin in Skin. *Cell*.

[54] Yamamoto H., Flannery M. L., Kupriyanov S. (1998). Defective Trophoblast Function in Mice With a Targeted Mutation of Ets2. *Genes and Development*.

[55] Koch P. J., Mahoney M. G., Ishikawa H (1997). Targeted Disruption of the Pemphigus Vulgaris Antigen (Desmoglein 3) Gene in Mice Causes Loss of Keratinocyte Cell Adhesion With a Phenotype Similar to Pemphigus Vulgaris. *The Journal of Cell Biology*.

[56] Kljuic A., Bazzi H., Sundberg J. P (2003). Desmoglein 4 in Hair Follicle Differentiation and Epidermal Adhesion: Evidence From Inherited Hypotrichosis and Acquired Pemphigus Vulgaris. *Cell*.

[57] Cotsarelis G., Millar S. E. (2001). Towards a Molecular Understanding of Hair Loss and Its Treatment. *Trends in Molecular Medicine*.

[58] Kesika P., Sivamaruthi B. S., Thangaleela S., Bharathi M., Chaiyasut C. (2023). Role and Mechanisms of Phytochemicals in Hair Growth and Health. *Pharmaceuticals*.

[59] Ji S., Zhu Z., Sun X., Fu X. (2021). Functional Hair Follicle Regeneration: An Updated Review. *Signal Transduction and Targeted Therapy*.

[60] Choi B. Y. (2020). Targeting Wnt/*β*-Catenin Pathway for Developing Therapies for Hair Loss. *International Journal of Molecular Sciences*.

[61] Hussain S. A. I., Sen B., Das Gupta A., Mandal U. K. (2020). Novel Multi-Objective Decision-Making and Trade-Off Approach for Selecting Optimal Machining Parameters of Inconel-800 Superalloy. *Arabian Journal for Science and Engineering*.

[62] Bhowmik A., Meher A., Biswas S. (2022). Synthesis and Characterization of Borosilicate Glass Powder-Reinforced Novel Lightweight Aluminum Matrix Composites. *Advances in Materials Science and Engineering*.

[63] Gentile P., Garcovich S. (2019). Advances in Regenerative Stem Cell Therapy in Androgenic Alopecia and Hair Loss: Wnt Pathway, Growth-Factor, and Mesenchymal Stem Cell Signaling Impact Analysis on Cell Growth and Hair Follicle Development. *Cells*.

[64] Shimizu Y., Ntege E. H., Sunami H., Inoue Y. (2022). Regenerative Medicine Strategies for Hair Growth and Regeneration: A Narrative Review of Literature. *Regenerative Therapy*.

[65] Pensato R., Al-Amer R., La Padula S. (2024). Review of Human Hair Follicle Biology: Dynamics of Niches and Stem Cell Regulation for Possible Therapeutic Hair Stimulation for Plastic Surgeons. *Aesthetic Plastic Surgery*.

[66] Plotczyk M., Jimenez F., Jimenez F., Higgins C., Hair F. R. (2022). Hair Follicles in Wound Healing and Skin Remodelling. *Stem Cell Biology and Regenerative Medicine*.

[67] Sadgrove N. J., Simmonds M. S. J. (2021). Topical and Nutricosmetic Products for Healthy Hair and Dermal Antiaging Using “Dual‐Acting” (2 for 1) Plant‐Based Peptides, Hormones, and Cannabinoidsdual-Acting (2 for 1) Plant-Based Peptides, Hormones, and Cannabinoids. *FASEB BioAdvances*.

[68] Sen B., Hussain S. A. I., Gupta M. K., Mia M., Mandal U. K. (2021). Swarm Intelligence Based Selection of Optimal End-Milling Parameters Under Minimum Quantity Nano-Green Lubricating Environment. *Proceedings of the Institution of Mechanical Engineers—Part C: Journal of Mechanical Engineering Science*.

[69] Gokce N., Basgoz N., Kenanoglu S. (2022). An Overview of the Genetic Aspects of Hair Loss and Its Connection With Nutrition. *Journal of Preventive Medicine and Hygiene*.

[70] Saha D., Gurung J., Roy B., Pulikkal A. K., Bhowmik A., Pattanayak S. (2022). Optimizing Pyrolysis Process Parameters of Plastic Grocery Bag, With Mass–Energy Assessment and Characterization of Oil at Optimal Condition. *Clean Technologies and Environmental Policy*.

[71] Sen B., Yadav S. K., Kumar G., Mukhopadhyay P., Ghosh S. (2023). Performance of Eco-Benign Lubricating/Cooling Mediums in Machining of Superalloys: A Comprehensive Review From the Perspective of Triple Bottom Line Theory. *Sustainable Materials and Technologies*.

[72] Castro A. R., Logarinho E. (2020). Tissue Engineering Strategies for Human Hair Follicle Regeneration: How Far From a Hairy Goal?. *Stem Cells Translational Medicine*.

